# Poly[[(μ-2,2′-bipyrimidine-κ^4^ *N* ^1^,*N* ^1′^:*N* ^3^,*N* ^3′^)(μ-sulfato-κ^2^ *O*:*O*′)zinc(II)] monohydrate]

**DOI:** 10.1107/S1600536810001649

**Published:** 2010-01-30

**Authors:** Aaron Oxendine, Jennifer Kelley, LeRoy Peterson, Mark D. Smith, Hans-Conrad zur Loye

**Affiliations:** a Chemistry Department, Francis Marion University, Florence, South Carolina 29502, USA; b Department of Chemistry and Biochemistry, University of South Carolina, Columbia, South Carolina 29208, USA

## Abstract

In the title compound, {[Zn(SO_4_)(C_8_H_6_N_4_)]·H_2_O}_*n*_, the Zn^II^ atom is in a distorted octa­hedral environment. The Zn^II^ atoms are bridged by both 2,2′-bipyrimidine and sulfate ligands, thus forming a three-dimensional polymeric metal–organic solid that contains uncoordinated water mol­ecules in the inter­stitial space. O—H⋯O hydrogen bonding consolidates the crystal structure.

## Related literature

For general background to metal-organic polymers with 2,2′-bipyrimidine ligands, see: De Munno *et al.* (1995 ▶); Kawata *et al.* (1998 ▶); Marshall *et al.* (2000 ▶); Wang *et al.* (2007 ▶). For a related structure, see: De Munno & Julve (1994 ▶).
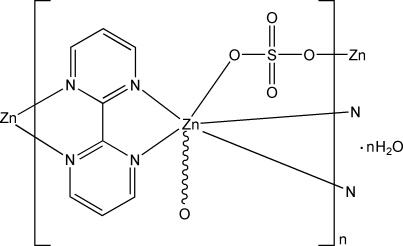


## Experimental

### 

#### Crystal data

[Zn(SO_4_)(C_8_H_6_N_4_)]·H_2_O
*M* *_r_* = 337.61
Monoclinic, 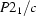

*a* = 8.9935 (3) Å
*b* = 13.9783 (5) Å
*c* = 9.8459 (4) Å
β = 117.007 (1)°
*V* = 1102.79 (7) Å^3^
*Z* = 4
Mo *K*α radiation
μ = 2.44 mm^−1^
*T* = 294 K
0.20 × 0.15 × 0.08 mm

#### Data collection

Bruker SMART APEX CCD diffractometer
Absorption correction: multi-scan (*SADABS*; Bruker, 2001 ▶) *T*_min_ = 0.874, *T*_max_ = 1.000
12167 measured reflections
2254 independent reflections
2087 reflections with *I* > 2σ(*I*)
*R*_int_ = 0.030

#### Refinement

*R*[*F* ^2^ > 2σ(*F* ^2^)] = 0.023
*wR*(*F* ^2^) = 0.063
*S* = 1.05
2254 reflections
180 parameters
1 restraint
H atoms treated by a mixture of independent and constrained refinement
Δρ_max_ = 0.40 e Å^−3^
Δρ_min_ = −0.52 e Å^−3^

### 

Data collection: *SMART* (Bruker, 2007 ▶); cell refinement: *SAINT* (Bruker, 2007 ▶); data reduction: *SAINT*; program(s) used to solve structure: *SHELXS97* (Sheldrick, 2008 ▶); program(s) used to refine structure: *SHELXL97* (Sheldrick, 2008 ▶); molecular graphics: *DIAMOND* (Brandenburg, 1999 ▶); software used to prepare material for publication: *SHELXTL* (Sheldrick, 2008 ▶).

## Supplementary Material

Crystal structure: contains datablocks global, I. DOI: 10.1107/S1600536810001649/hy2264sup1.cif

Structure factors: contains datablocks I. DOI: 10.1107/S1600536810001649/hy2264Isup2.hkl

Additional supplementary materials:  crystallographic information; 3D view; checkCIF report

## Figures and Tables

**Table 1:** Selected bond lengths (Å)

Zn1—N1	2.2646 (16)
Zn1—N2^i^	2.1228 (15)
Zn1—N3	2.1403 (17)
Zn1—N4^ii^	2.2852 (16)
Zn1—O1	2.0302 (14)
Zn1—O2^iii^	2.0371 (14)

Symmetry codes: (i) 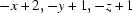; (ii) 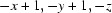; (iii) 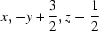.

**Table 2:** Hydrogen-bond geometry (Å, °)

*D*—H⋯*A*	*D*—H	H⋯*A*	*D*⋯*A*	*D*—H⋯*A*
O5—H5*A*⋯O3	0.78 (2)	2.07 (2)	2.838 (3)	166 (3)
O5—H5*B*⋯O2^iv^	0.78 (2)	2.11 (2)	2.883 (2)	173 (3)

Symmetry code: (iv) 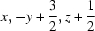.

**Fig. 1.**
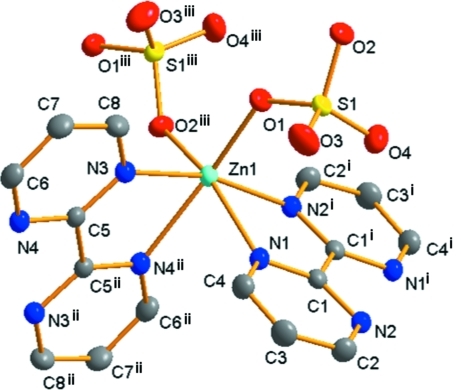
Coordination environment of ZnII atom in (I). Displacement ellipsoids are shown at the 50% probability level. H atoms and water molecule have been omitted for clarity. [Symmetry codes: (i) 2-x, 1-y, 1-z; (ii) 1-x, 1-y, -z; (iii) x, 3/2-y, -1/2+z.]

**Fig. 2.**
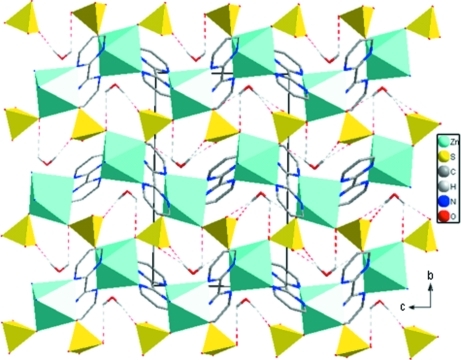
Polyhedral and wireframe representation of the crystal packing in (I). All H atoms except those of water have been omitted for clarity. Hydrogen bonds are represented by dashed lines.

## supplementary crystallographic information

### Comment

Metal-organic polymers utilizing the 2,2'-bipyrimidine (bpm) ligand are being studied due to the ability of bpm to produce interesting and potentially useful materials (Kawata *et al.*, 1998; Marshall *et al.*, 2000; Wang *et al.*, 2007). Such features are often associated with the ability of this ligand to link metal centers through the bis-bidentate coordination mode (De Munno *et al.*, 1995). Herein we report the crystal structure of the title compound, (I), that is a three-dimensional metal-organic framework where bpm binds Zn^II^ atoms in this fashion.

The crystal structure of (I), which incidentally is isostructural with [Cu(bpm)(SO_4_)].H_2_O (Kawata *et al.*, 1998), is a three-dimensional polymeric solid with an asymmetric unit consisting of one Zn^II^ atom, two half-bpm ligands, a sulfate ligand, and one lattice water. The Zn^II^ atom resides in a distorted octahedral environment composed of four N donors from a pair of equivalent bpm ligands, and two O atoms from two equivalent sulfate anions (Fig. 1). All of the Zn—N and Zn—O bond distances are in a normal range (Table 1).

The bpm ligand bridges Zn^II^ atoms in a bis-bidentate fashion, producing undulating chains running along the [101] direction. Further, the sulfate ligand serves to bridge neighboring chains, thus forming a three-dimensional microporous solid. The pores are occupied by lattice waters that are hydrogen bonded to uncoordinated O2 and O3 atoms of nearby sulfate anions (Table 2 and Fig. 2).

It is also interesting to note that the crystal structure of (I) differs from that of [Zn_2_(µ-bpm)(H_2_O)_8_](SO_4_)_2_.2H_2_O (II) (De Munno & Julve, 1994), which contains the same chemical components as (I), but was synthesized under different synthetic conditions (see below).

### Experimental

All starting chemicals were purchased from commercial sources and used as received. An aqueous solution of zinc sulfate heptahydrate (0.10 mmol, 10 ml) was slowly added to 10 ml of an ethanolic solution composed of bpm (0.050 mmol) and 4,4'-bipyridine (bpy) (0.050 mmol). Colorless, plate-like crystals formed within several weeks after slow evaporation of all the solvent under ambient conditions. Although bpy was not incorporated into the crystal structure of (I), it was required for synthesis of the crystalline product, as no such crystals were formed without it with all other conditions being the same. The synthesis in water alone using only zinc sulfate heptahydrate and bpm was reported to produce (II), as previously mentioned.

### Refinement

H atoms bonded to C atoms were placed in geometrically idealized positions and refined as riding atoms, with C—H = 0.93 Å and *U*_iso_(H) = 1.2*U*_eq_(C). H atoms of water molecule were located from a difference Fourier map and refined isotropically, with their O—H distances restrained to 0.84 (2) Å.

### Crystal data

**Table:**

[Zn(SO_4_)(C_8_H_6_N_4_)]·H_2_O	*F*(000) = 680
*M**_r_* = 337.61	*D*_x_ = 2.033 Mg m^−^^3^
Monoclinic, *P*2_1_/*c*	Mo *K*α radiation, λ = 0.71073 Å
Hall symbol: -P 2ybc	Cell parameters from 6452 reflections
*a* = 8.9935 (3) Å	θ = 2.5–26.4°
*b* = 13.9783 (5) Å	µ = 2.44 mm^−^^1^
*c* = 9.8459 (4) Å	*T* = 294 K
β = 117.007 (1)°	Prism, colorless
*V* = 1102.79 (7) Å^3^	0.20 × 0.15 × 0.08 mm
*Z* = 4	

### Data collection

**Table:**

Bruker SMART APEX CCD diffractometer	2254 independent reflections
Radiation source: fine-focus sealed tube	2087 reflections with *I* > 2σ(*I*)
graphite	*R*_int_ = 0.030
φ and ω scans	θ_max_ = 26.4°, θ_min_ = 2.5°
Absorption correction: multi-scan (*SADABS*; Bruker, 2001)	*h* = −11→11
*T*_min_ = 0.874, *T*_max_ = 1.000	*k* = −17→17
12167 measured reflections	*l* = −12→12

### Refinement

**Table:**

Refinement on *F*^2^	Primary atom site location: structure-invariant direct methods
Least-squares matrix: full	Secondary atom site location: difference Fourier map
*R*[*F*^2^ > 2σ(*F*^2^)] = 0.023	Hydrogen site location: mixed
*wR*(*F*^2^) = 0.063	H atoms treated by a mixture of independent and constrained refinement
*S* = 1.05	*w* = 1/[σ^2^(*F*_o_^2^) + (0.0366*P*)^2^ + 0.505*P*] where *P* = (*F*_o_^2^ + 2*F*_c_^2^)/3
2254 reflections	(Δ/σ)_max_ = 0.001
180 parameters	Δρ_max_ = 0.40 e Å^−^^3^
1 restraint	Δρ_min_ = −0.52 e Å^−^^3^

### Special details

**Table:**

Refinement. Water molecule O—H bonds restrained to be approximately equal.

### Fractional atomic coordinates and isotropic or equivalent isotropic displacement parameters (Å^2^)

**Table:**

	*x*	*y*	*z*	*U*_iso_*/*U*_eq_	
Zn1	0.77696 (3)	0.609668 (15)	0.23367 (2)	0.01838 (9)	
S1	0.81536 (6)	0.72465 (3)	0.52650 (5)	0.01914 (12)	
C1	0.9291 (2)	0.47924 (13)	0.5096 (2)	0.0177 (4)	
C2	0.8397 (3)	0.38425 (14)	0.6454 (2)	0.0243 (4)	
H2	0.8615	0.3449	0.7285	0.029*	
C3	0.6764 (3)	0.40060 (15)	0.5389 (3)	0.0268 (5)	
H3	0.5878	0.3722	0.5478	0.032*	
C4	0.6501 (2)	0.46036 (15)	0.4197 (2)	0.0243 (4)	
H4	0.5412	0.4735	0.3478	0.029*	
C5	0.4422 (2)	0.53564 (13)	0.0071 (2)	0.0180 (4)	
C6	0.1759 (2)	0.58714 (15)	−0.0698 (2)	0.0247 (4)	
H6	0.0615	0.5802	−0.1296	0.030*	
C7	0.2350 (3)	0.66300 (16)	0.0306 (2)	0.0274 (4)	
H7	0.1626	0.7078	0.0381	0.033*	
C8	0.4051 (3)	0.66992 (15)	0.1193 (2)	0.0261 (4)	
H8	0.4480	0.7202	0.1882	0.031*	
N1	0.77690 (19)	0.50046 (12)	0.40355 (18)	0.0195 (3)	
N2	0.96733 (19)	0.42423 (12)	0.63088 (18)	0.0185 (3)	
N3	0.5106 (2)	0.60557 (11)	0.10833 (19)	0.0210 (4)	
N4	0.2798 (2)	0.52321 (12)	−0.08307 (18)	0.0203 (3)	
O1	0.78154 (19)	0.72359 (10)	0.36342 (16)	0.0271 (3)	
O2	0.81664 (19)	0.82843 (10)	0.56562 (16)	0.0263 (3)	
O3	0.6819 (2)	0.67544 (12)	0.54284 (19)	0.0374 (4)	
O4	0.97797 (19)	0.68377 (12)	0.62188 (17)	0.0337 (4)	
O5	0.7510 (2)	0.55805 (14)	0.7998 (2)	0.0384 (4)	
H5A	0.747 (3)	0.5945 (17)	0.738 (3)	0.034 (8)*	
H5B	0.769 (4)	0.5931 (19)	0.867 (3)	0.046 (9)*	

### Atomic displacement parameters (Å^2^)

**Table:**

	*U*^11^	*U*^22^	*U*^33^	*U*^12^	*U*^13^	*U*^23^
Zn1	0.01726 (14)	0.01966 (14)	0.01514 (13)	−0.00064 (8)	0.00468 (10)	0.00031 (8)
S1	0.0218 (2)	0.0193 (2)	0.0160 (2)	−0.00226 (18)	0.00831 (19)	−0.00235 (18)
C1	0.0188 (9)	0.0163 (9)	0.0171 (9)	0.0000 (7)	0.0073 (7)	−0.0026 (7)
C2	0.0248 (10)	0.0255 (11)	0.0250 (10)	−0.0019 (8)	0.0135 (9)	0.0039 (8)
C3	0.0202 (10)	0.0308 (12)	0.0317 (11)	−0.0050 (8)	0.0138 (9)	−0.0002 (9)
C4	0.0169 (9)	0.0268 (11)	0.0256 (10)	0.0000 (8)	0.0066 (8)	−0.0002 (8)
C5	0.0192 (9)	0.0183 (9)	0.0155 (9)	0.0007 (7)	0.0071 (7)	0.0018 (7)
C6	0.0184 (9)	0.0281 (11)	0.0252 (10)	0.0034 (8)	0.0079 (8)	0.0051 (9)
C7	0.0266 (10)	0.0267 (11)	0.0306 (11)	0.0079 (8)	0.0145 (9)	0.0026 (9)
C8	0.0304 (11)	0.0213 (10)	0.0254 (10)	0.0026 (8)	0.0116 (9)	−0.0033 (8)
N1	0.0160 (7)	0.0211 (8)	0.0185 (8)	0.0004 (6)	0.0053 (6)	0.0003 (7)
N2	0.0180 (8)	0.0199 (8)	0.0171 (8)	−0.0005 (6)	0.0075 (6)	−0.0003 (6)
N3	0.0203 (8)	0.0204 (9)	0.0195 (8)	0.0003 (6)	0.0066 (7)	−0.0006 (6)
N4	0.0180 (7)	0.0218 (8)	0.0189 (8)	−0.0007 (6)	0.0064 (6)	0.0013 (6)
O1	0.0408 (9)	0.0226 (8)	0.0170 (7)	0.0002 (6)	0.0124 (6)	−0.0035 (6)
O2	0.0393 (8)	0.0204 (7)	0.0207 (7)	−0.0009 (6)	0.0150 (6)	−0.0040 (6)
O3	0.0378 (9)	0.0417 (10)	0.0404 (9)	−0.0170 (7)	0.0245 (8)	−0.0086 (8)
O4	0.0314 (8)	0.0363 (9)	0.0263 (8)	0.0089 (7)	0.0067 (7)	−0.0002 (7)
O5	0.0487 (11)	0.0327 (10)	0.0320 (9)	−0.0069 (8)	0.0167 (8)	−0.0042 (8)

### Geometric parameters (Å, °)

**Table:**

Zn1—N1	2.2646 (16)	C3—C4	1.371 (3)
Zn1—N2^i^	2.1228 (15)	C3—H3	0.9300
Zn1—N3	2.1403 (17)	C4—N1	1.343 (3)
Zn1—N4^ii^	2.2852 (16)	C4—H4	0.9300
Zn1—O1	2.0302 (14)	C5—N3	1.331 (2)
Zn1—O2^iii^	2.0371 (14)	C5—N4	1.332 (2)
S1—O4	1.4491 (15)	C5—C5^ii^	1.492 (4)
S1—O3	1.4547 (15)	C6—N4	1.341 (3)
S1—O1	1.4924 (14)	C6—C7	1.381 (3)
S1—O2	1.4996 (15)	C6—H6	0.9300
C1—N1	1.324 (2)	C7—C8	1.378 (3)
C1—N2	1.327 (2)	C7—H7	0.9300
C1—C1^i^	1.489 (4)	C8—N3	1.346 (3)
C2—N2	1.341 (3)	C8—H8	0.9300
C2—C3	1.382 (3)	O5—H5A	0.78 (2)
C2—H2	0.9300	O5—H5B	0.78 (2)
			
O1—Zn1—O2^iii^	102.53 (6)	N1—C4—C3	121.99 (18)
O1—Zn1—N2^i^	94.20 (6)	N1—C4—H4	119.0
O2^iii^—Zn1—N2^i^	93.77 (6)	C3—C4—H4	119.0
O1—Zn1—N3	94.66 (6)	N3—C5—N4	126.10 (17)
O2^iii^—Zn1—N3	96.09 (6)	N3—C5—C5^ii^	117.1 (2)
N2^i^—Zn1—N3	165.00 (6)	N4—C5—C5^ii^	116.8 (2)
O1—Zn1—N1	94.05 (6)	N4—C6—C7	121.50 (18)
O2^iii^—Zn1—N1	160.92 (6)	N4—C6—H6	119.2
N2^i^—Zn1—N1	75.46 (6)	C7—C6—H6	119.2
N3—Zn1—N1	91.85 (6)	C8—C7—C6	117.60 (18)
O1—Zn1—N4^ii^	168.69 (6)	C8—C7—H7	121.2
O2^iii^—Zn1—N4^ii^	83.58 (6)	C6—C7—H7	121.2
N2^i^—Zn1—N4^ii^	94.89 (6)	N3—C8—C7	121.46 (19)
N3—Zn1—N4^ii^	75.07 (6)	N3—C8—H8	119.3
N1—Zn1—N4^ii^	81.74 (6)	C7—C8—H8	119.3
O4—S1—O3	112.49 (10)	C1—N1—C4	116.24 (17)
O4—S1—O1	110.24 (9)	C1—N1—Zn1	112.75 (12)
O3—S1—O1	109.82 (9)	C4—N1—Zn1	130.81 (13)
O4—S1—O2	109.10 (9)	C1—N2—C2	116.91 (16)
O3—S1—O2	109.85 (9)	C1—N2—Zn1^i^	117.30 (12)
O1—S1—O2	105.07 (8)	C2—N2—Zn1^i^	125.12 (14)
N1—C1—N2	126.29 (17)	C5—N3—C8	116.61 (17)
N1—C1—C1^i^	116.8 (2)	C5—N3—Zn1	117.85 (13)
N2—C1—C1^i^	117.0 (2)	C8—N3—Zn1	125.50 (14)
N2—C2—C3	121.07 (19)	C5—N4—C6	116.72 (17)
N2—C2—H2	119.5	C5—N4—Zn1^ii^	113.14 (12)
C3—C2—H2	119.5	C6—N4—Zn1^ii^	130.12 (14)
C4—C3—C2	117.47 (18)	S1—O1—Zn1	128.37 (9)
C4—C3—H3	121.3	S1—O2—Zn1^iv^	129.47 (9)
C2—C3—H3	121.3	H5A—O5—H5B	100 (3)
			
N2—C2—C3—C4	−1.1 (3)	C7—C8—N3—C5	0.4 (3)
C2—C3—C4—N1	1.4 (3)	C7—C8—N3—Zn1	178.11 (15)
N4—C6—C7—C8	−1.2 (3)	O1—Zn1—N3—C5	−176.93 (14)
C6—C7—C8—N3	0.4 (3)	O2^iii^—Zn1—N3—C5	79.91 (14)
N2—C1—N1—C4	−1.5 (3)	N2^i^—Zn1—N3—C5	−50.9 (3)
C1^i^—C1—N1—C4	178.1 (2)	N1—Zn1—N3—C5	−82.71 (14)
N2—C1—N1—Zn1	173.85 (15)	N4^ii^—Zn1—N3—C5	−1.76 (13)
C1^i^—C1—N1—Zn1	−6.5 (3)	O1—Zn1—N3—C8	5.40 (17)
C3—C4—N1—C1	−0.2 (3)	O2^iii^—Zn1—N3—C8	−97.75 (17)
C3—C4—N1—Zn1	−174.55 (15)	N2^i^—Zn1—N3—C8	131.4 (2)
O1—Zn1—N1—C1	−85.87 (13)	N1—Zn1—N3—C8	99.63 (17)
O2^iii^—Zn1—N1—C1	64.6 (2)	N4^ii^—Zn1—N3—C8	−179.43 (18)
N2^i^—Zn1—N1—C1	7.42 (12)	N3—C5—N4—C6	−0.3 (3)
N3—Zn1—N1—C1	179.32 (13)	C5^ii^—C5—N4—C6	179.8 (2)
N4^ii^—Zn1—N1—C1	104.70 (13)	N3—C5—N4—Zn1^ii^	−178.55 (15)
O1—Zn1—N1—C4	88.67 (18)	C5^ii^—C5—N4—Zn1^ii^	1.5 (2)
O2^iii^—Zn1—N1—C4	−120.8 (2)	C7—C6—N4—C5	1.1 (3)
N2^i^—Zn1—N1—C4	−178.04 (18)	C7—C6—N4—Zn1^ii^	179.05 (15)
N3—Zn1—N1—C4	−6.14 (18)	O4—S1—O1—Zn1	57.96 (14)
N4^ii^—Zn1—N1—C4	−80.76 (18)	O3—S1—O1—Zn1	−66.53 (14)
N1—C1—N2—C2	1.8 (3)	O2—S1—O1—Zn1	175.38 (10)
C1^i^—C1—N2—C2	−177.8 (2)	O2^iii^—Zn1—O1—S1	−158.01 (11)
N1—C1—N2—Zn1^i^	172.93 (15)	N2^i^—Zn1—O1—S1	−63.20 (12)
C1^i^—C1—N2—Zn1^i^	−6.7 (3)	N3—Zn1—O1—S1	104.68 (12)
C3—C2—N2—C1	−0.4 (3)	N1—Zn1—O1—S1	12.49 (12)
C3—C2—N2—Zn1^i^	−170.74 (15)	N4^ii^—Zn1—O1—S1	80.2 (3)
N4—C5—N3—C8	−0.5 (3)	O4—S1—O2—Zn1^iv^	−84.54 (13)
C5^ii^—C5—N3—C8	179.5 (2)	O3—S1—O2—Zn1^iv^	39.20 (15)
N4—C5—N3—Zn1	−178.36 (14)	O1—S1—O2—Zn1^iv^	157.27 (10)
C5^ii^—C5—N3—Zn1	1.6 (3)		

Symmetry codes: (i) −*x*+2, −*y*+1, −*z*+1; (ii) −*x*+1, −*y*+1, −*z*; (iii) *x*, −*y*+3/2, *z*−1/2; (iv) *x*, −*y*+3/2, *z*+1/2.

### Hydrogen-bond geometry (Å, °)

**Table:**

*D*—H···*A*	*D*—H	H···*A*	*D*···*A*	*D*—H···*A*
O5—H5A···O3	0.78 (2)	2.07 (2)	2.838 (3)	166 (3)
O5—H5B···O2^iv^	0.78 (2)	2.11 (2)	2.883 (2)	173 (3)

Symmetry codes: (iv) *x*, −*y*+3/2, *z*+1/2.
